# Defining risk variables causing gas embolism in loggerhead sea turtles (*Caretta caretta*) caught in trawls and gillnets

**DOI:** 10.1038/s41598-017-02819-5

**Published:** 2017-06-01

**Authors:** Andreas Fahlman, Jose Luis Crespo-Picazo, Blair Sterba-Boatwright, Brian A. Stacy, Daniel Garcia-Parraga

**Affiliations:** 1 Fundación Oceanogràfic de la Comunidad Valenciana, Gran Vía Marqués del Turia 19, 46005 Valencia, Spain; 2 Texas A & M University-Corpus Christi, 6300 Ocean Drive, Corpus Christi, TX 78412 USA; 3 National Marine Fisheries Service, Office of Protected Resources, University of Florida, College of Veterinary Medicine (duty station), Post Office Box 110885, Gainesville, FL 32611 USA

## Abstract

Incidental capture, or ‘bycatch’ in fishing gear is a major global threat to sea turtle populations. A recent study showed that underwater entrapment in fishing gear followed by rapid decompression may cause gas bubble formation within the blood stream (embolism) and tissues leading to organ injury, impairment, and even mortality in some bycaught individuals. We analyzed data from 128 capture events using logistic and ordinal regression to examine risk factors associated with gas embolism in sea turtles captured in trawls and gillnets. Likelihood of fatal decompression increases with increasing depth of gear deployment. A direct relationship was found between depth, risk and severity of embolism, which has not been previously demonstrated in any breath-hold diving species. For the trawl fishery in this study, an average trawl depth of 65 m was estimated to result in 50% mortality in by-caught turtles throughout the year. This finding is critical for a more accurate estimation of sea turtle mortality rates resulting from different fisheries and for devising efforts to avoid or minimize the harmful effects of capture.

## Introduction

Decompression sickness (DCS) is a disorder that is mostly reported in association with scuba diving in humans. As divers descend, increasing pressure results in increased solubility of gases within the body, and the blood and tissues absorb greater concentrations of pulmonary gas. When the blood or tissue tension exceeds ambient pressure during the ascent or decompression phase of the dive, gas may come out of solution and form bubbles within the blood stream and tissues, especially if the reduction in pressure is too fast^1^. If sufficiently severe, gas bubble formation or gas embolism (GE) causes clinical symptoms and tissue damage known as DCS^2^. Although bubbles can form from any gas under certain dive profiles, N_2_ bubbles are the most common and occur when atmospheric air is breathed during diving. In air-breathing terrestrial mammals, the risk of DCS correlates with dive depth (pressure), time at depth (dive duration), ascent rate (pressure reduction), temperature^3^, and allometric variation in cardiac output^1, 4, 5^.

Breath-hold diving vertebrates, such as marine mammals, turtles and penguins, have numerous physiological, anatomical and behavioral traits that allow them to efficiently hunt for prey underwater while avoiding pressure related problems like GE. These traits help alter gas exchange to avoid excessive blood and tissue N_2_ tension, thereby minimizing risk of DCS^6, 7^. Until recently, it was thought that breath-hold diving vertebrates do not experience DCS as they retain a limited amount of N_2_ in their lungs or minimize N_2_ uptake during each dive^8^. However, it has been suggested that N_2_ could accumulate in the blood and tissues during repeated dives, or as a result of disruption of protective physiological or behavioral mechanisms, making DCS more likely under certain circumstances^9, 10^. For example, increased blood flow, as may happen during stress or if an animal is trying to escape, has been shown to correlate with DCS risk in terrestrial mammals^1^. In cetaceans, theoretical models suggest that behavioral (e.g. increased ascent, prolonged dive duration) or physiological (e.g. increased blood flow, elevated CO_2_) alterations associated with exposure to sonar may increase the blood and tissue N_2_ levels and thereby the risk of DCS^9, 11, 12^.

At the beginning of this century, necropsy findings observed in stranded cetaceans were found to be consistent with DCS-like disease^13^. Additional work since these discoveries suggests that marine mammals live with elevated concentrations of N_2_ that may cause asymptomatic GE during natural dives, but potentially cause DCS under unusual circumstances^9, 10, 14, 15^. However, whether or not air-breathing marine vertebrates experience DCS as it is classically defined has been quite controversial^2, 10^. In 2014, a study of bycaught loggerhead turtles (*Caretta caretta*) entrapped at depth in trawls and gillnets unquestionably demonstrated that breath hold-diving vertebrates develop GE^16^. These turtles were shown to have blood and tissue GE associated with the presence of clinical symptoms that were resolved by recompression therapy, thus demonstrating evidence-based diagnosis of DCS^2^.

Notably, sea turtles that develop clinical signs of DCS associated with GE sometimes are in seemingly good health upon initial capture by fishing vessels, but their condition deteriorates over hours after surfacing. The common practice in many fisheries worldwide is to directly release live and alert bycaught turtles upon capture^17–19^, thus these delayed effects may go unobserved. Undetected mortality following release, also referred to as post-release mortality, may lead to significant underestimates of the numbers of turtles killed by fisheries interactions.

To date, GE and resulting DCS primarily have been studied in loggerhead turtles bycaught in trawls and gillnets in the Mediterranean Sea^16^. Intravascular bubbles have also been detected in a leatherback turtle (*Dermochelys coriacea*) caught by trawling and in a green turtle (*Chelonia mydas*) captured by gillnet, indicating that other species are also susceptible (Crespo-Picazo and Garcia-Parraga, unpublished observation). In addition, the pathophysiological mechanism of GE is unlikely to be unique to fisheries studied to date. Thus, evidence suggests any sea turtle captured underwater and brought to the surface under certain conditions may be at risk of DCS. The capture conditions and biological factors that may lead to DCS in sea turtles, however, have not been explored.

In the current study, we use available data collected opportunistically from loggerhead turtles bycaught in trawls and gillnets and diagnosed with various degrees of GE to investigate potential variables associated with developing this condition. We specifically examined capture depth (pressure), temperature, and body size, variables known to correlate with DCS in other taxa^1^. While a number of other variables, such as water temperature at depth, dive duration, ascent rate, and the time elapsed from surfacing until treatment may alter risk and outcome^1, 3^, these data were not available in the current study.

## Results

**Figure 1:**
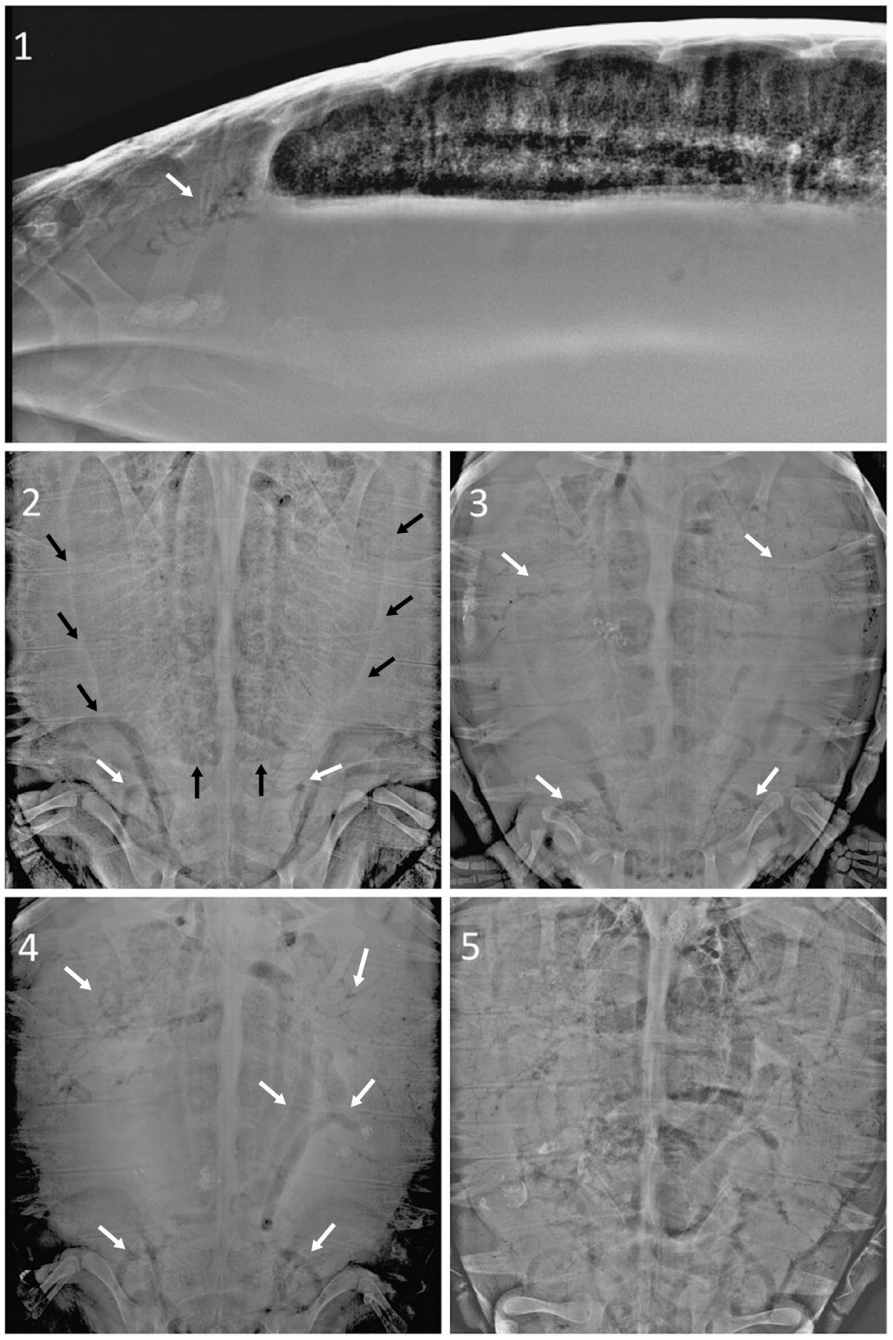
Examples of different degrees of gas embolism (GE) demonstrating the scoring criteria used in this study. Gas emboli are detected as radiolucent (black) anomalies following blood vessels (white arrowheads). Lung shape and contrast are also reduced (pulmonary silhouette indicated by black arrowheads) in dorsal-ventral (DV) radiographs as pulmonary parenchyma becomes collapsed by gas accumulation and expansion of other coelomic organs. Score (1) very mild GE within the kidneys in a lateral-lateral radiograph. Score (2) mild GE is clearly visible within the kidneys in a dorsal-ventral (DV) radiograph. Score (3) moderate GE is evident within both the kidneys and some peripheral hepatic vessels. Note that the reduced pulmonary silhouette compared to (2). Score (4) moderate-severe GE is observed within some larger blood vessels, including hepatic vessels and precavas, as well as the kidneys. The lung silhouette is further reduced. Score (5) abundant gas is present within the heart and principal veins (precavas, postcava, hepatic veins and portal-renal system), as well as many other peripheral areas and internal organs (including liver and kidney).

The overall incidence of GE in the 128 turtles included in the analyses was 55%. Descriptive statistics for parameters examined using our analyses, including severity of GE based on a scale of 1 to 5, are provided in Table 1. Examples of radiographs used to detect GE and characterize severity are shown in Fig. 1. Thirty percent of the study population had a GE score ≥ 3, which is often fatal without treatment^16^. There was no difference in the occurrence of GE, or GE score ≥ 3 when compared by gear type (Pearson Chi-square, χ^2^ = 2.32, P > 0.1). Both depth (1-way ANOVA, F_ratio_ = 156, P < 0.01) and the average duration that fishing gear was in the water (soak time, F_ratio_ = 17.9, P < 0.01) were significantly different between the gear types (Table 1).

**Table 1:** Numbers of examined bycaught sea turtles by gear type, detection of gas embolism (GE, shown in parentheses following total n examined), severity of GE based on a score of 1–5, and average curved carapace length (*CC*_L_), body mass (*M*_b_), sea surface temperature (SST), depth and estimated gear deployment time (with standard deviation and range). Superscripted values are number of animals.

Gear Type	n	GE score					*CC*_L_ (cm)	*M*_b_ (kg)	SST (°C)	Depth (m)	Soak time (hrs)
		1	2	3	4	5					
Gillnet	49 (23)	4	6	8	4	1	38 ± 8^49^ (25–61)	7.8 ± 3.7^48^ (2.1–27.8)	17 ± 3^49^ (13–25)	15 ± 9^38^ (3–50)	10.9 ± 3.7^18^ (1.3–19.5)
Trawl	79 (49)	10	13	12	10	4	46 ± 17^79^ (28–136)	14.7 ± 15.1^77^ (2.6–80.5)	15 ± 2^79^ (13–25)	55 ± 18^48^ (24–100)	2.9 ± 0.3^4^ (2.5–3.0)

A power regression showed that there was a significant relationship between body mass (*M*_b_) and curved carapace length (*CC*_L_, *M*_b_ = 8.88 × 10^−5^ × *C*_L_^3.09^, F_ratio_ = 7810, 123 df). Least square regression showed that larger animals, both by *M*_b_ and *CC*_L_, were caught at deeper depths (depth = 7.93 + 0.111 × *M*_b_: F_ratio_ = 3.89, 1 df, P = 0.05; depth = 37.2 + 0.139 × *CC*_L_: F_ratio_ = 4.32, 1 df, P < 0.05).

### Logistic regression

#### Trawl

**Figure 2:**
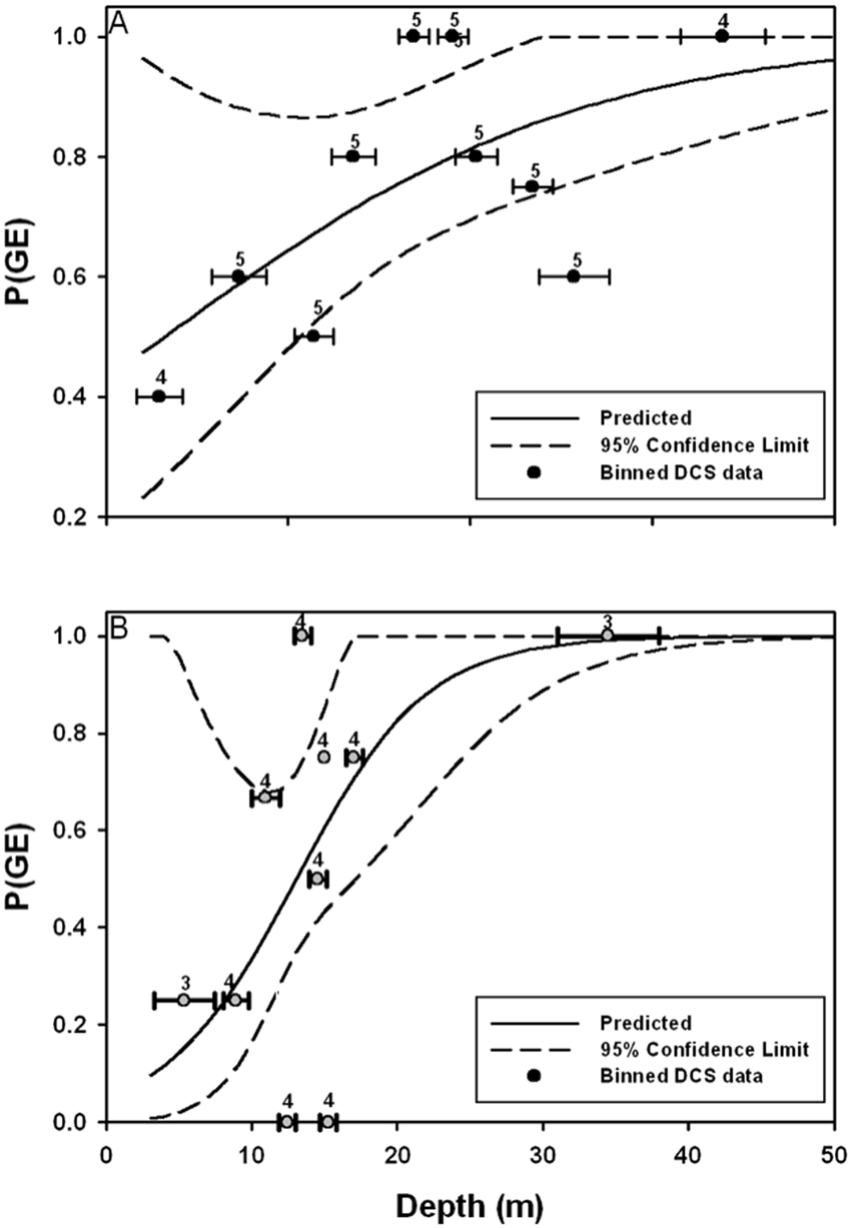
Model fit of the probability of gas emboli [P(GE)] by depth (±s.d) of gear deployment for turtles bycaught in (**A**) trawls and (**B**) gillnets. Number above symbol is number of animals in each bin.

From all variables studied, only fishing depth (χ^2^ = 4.56, df = 1, P < 0.05, Table 2) was important to explain the likelihood and severity of GE in turtles bycaught during trawling. Neither turtle *CC*_L_ (χ^2^ = 2.8, df = 1, P < 0.1), *M*_b_ (χ^2^ = 0.29, df = 1, P > 0.5), nor SST (χ^2^ = 0.14, df = 1, P > 0.7) correlated with GE score. Figure 2A shows the probability of GE [P(GE)] against trawl depth.

**Table 2:** Summary logistic regression results for parameters associated with gas embolism in bycaught turtles by gear type. β_0_ is intercept and β_1_ is the slope.

Gear type	Variable	β_0_	β_1_
Trawl	Depth	−1.164 ± 1.136	0.0439 ± 0.0222
Gillnet	Depth	−2.91 ± 1.48	0.222 ± 0.109
Gillnet	Sea Surface Temperature	−3.32 ± 1.89	0.197 ± 0.116

#### Gillnet

Fishing depth was important to explain the variation in GE risk in turtles bycaught in gillnets (χ^2^ = 8.13, df = 1, P < 0.01, Table 2, Fig. 2B). The slope of increasing occurrence of GE with depth was steeper for captures in gillnet as compared with trawl (Fig. 2A vs. B). In addition, there was a trend for SST to be important (χ^2^ = 3.26, df = 1, P < 0.1, Table 2), but this trend did not warrant inclusion in a multivariate model with depth (χ^2^ = 2.81, df = 1, P > 0.1, Table 2). Neither turtle *CC*_L_ (χ^2^ = 1.5, df = 1, P > 0.2) nor *M*_b_ (χ^2^ = 0.69, df = 1, P > 0.4) correlated with occurrence of GE.

### Ordinal regression

**Figure 3:**
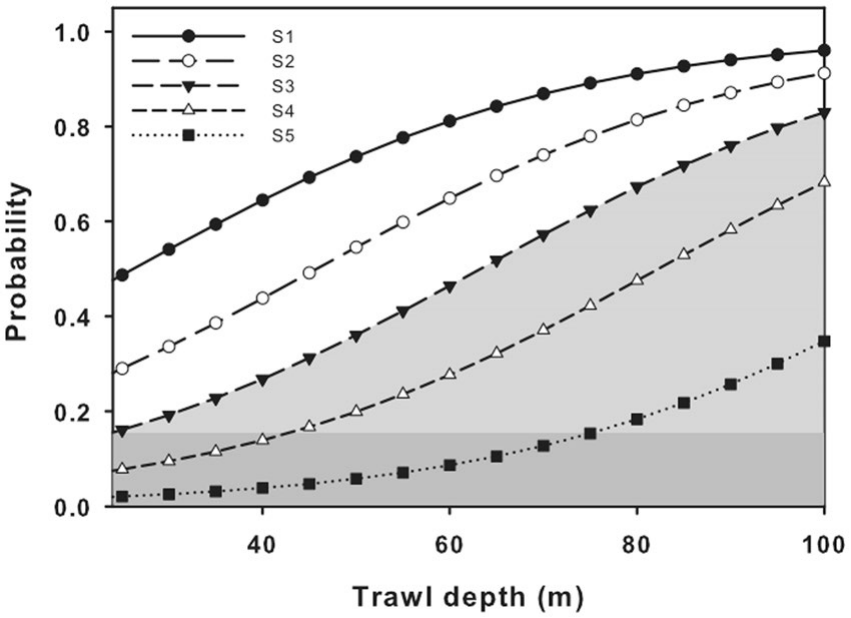
Model fit of the probability of gas embolism score (S1, very mild, to S5, severe) in bycaught turtles against trawling depth. Note that graph initiates at 20 m depth. The shaded gray area indicates expected mortalities in untreated turtles based on previously observed outcomes^16^.

**Figure 4:**
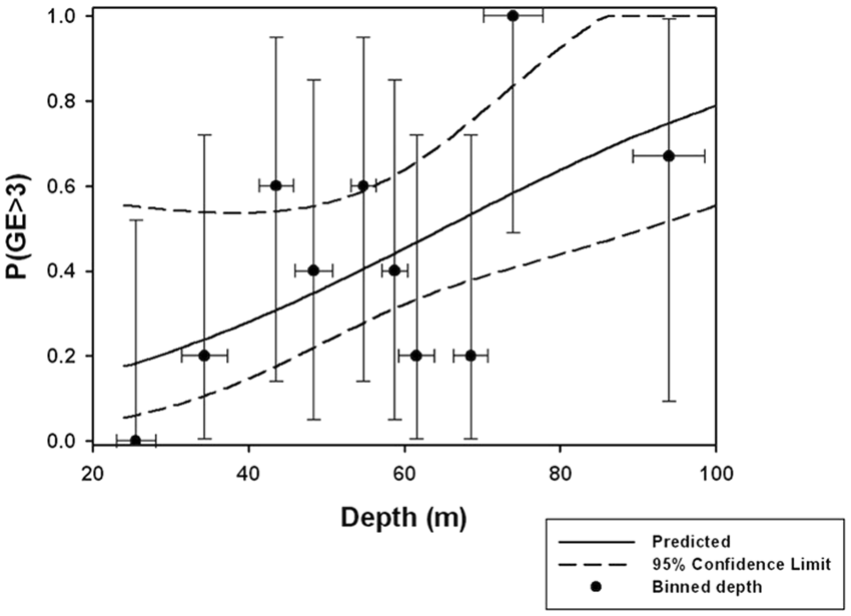
Model fit of the probability of fatal gas embolism (GE) in bycaught turtles against trawling depth. Data show risk of GE score equal to or higher than 3 (±95% binomial confidence limit) based on criteria used in this study for average depth (±s.d.) for 5 turtles in each bin. The solid line is data fitted to a logistic regression and the broken lines are the upper and lower 95% confidence limits.

Sufficient data were available to analyze conditions associated with GE score in sea turtles bycaught by trawls, but not for those captured in gillnets. Only fishing depth (χ^2^ = 7.52, df = 1, P < 0.01, Table 3) was important to explain the likelihood of an assigned GE score (Fig. 3). For the shallowest trawl depth (24 m), the risk of a very mild GE (score 1) was 20%, increasing to 98% at the deepest trawl depth of 100 m. The risk of the most severe GE (score 5) increased with depth to 35% at the deepest trawling depth. Considering that GE scores ≥ 3 are associated with fatal DCS, the average trawling depth resulting in 50% mortality, i.e. the probability for a GE score 3 or higher, for the trawling times used in the Valencian Community is estimated to be around 65 m (b_0_ = 2.45, b_1_ = 0.038, Fig. 4).

**Table 3:** Summary ordinal regression results for trawl bycaught turtles by severity of gas embolism based on a score (β_1–5_) of 0 (none) to 5 (severe).

Gear type	Variable	β_0_	β _1_	β _2_	β _3_	β _4_	β _5_
Trawl	Depth	−0.0431 ± 0.0162	−1.13 ± 0.89	−1.97 ± 0.92	−2.73 ± 0.95	−3.55 ± 1.00	−4.95 ± 1.13

## Discussion

Corresponding data for in-depth clinical evaluations and incidental captures of sea turtles in deepwater commercial fisheries are relatively difficult to obtain due to a number of logistical constraints. Although we were not able to examine the full suite of parameters known to correlate with DCS risk, the data presented in this study represents a large and unique collection of such information. While there are a number of limitations with data collected opportunistically, the analyses in this paper clearly show that the gear deployment depth significantly affects the likelihood of GE (Figs 2–4). For the fishing modalities in this region, 30% of examined bycaught turtles had a fatal degree of GE within a few hours of interaction. The global implications of the presented results are substantial as currently available information suggests that this risk is not limited to the species or specific fisheries examined in this study. Numbers of threatened or endangered sea turtles killed by incidental capture in commercial fisheries may be significantly underestimated if the effects of decompression are not considered.

Another parameter of considerable interest is the duration of submergence, which is determined by both length of gear deployment and the duration of an animal’s natural dive prior to capture. It is important to note that our study included trawls that fish deeper but for shorter duration as compared with gillnets, which are set shallower but are left submerged for longer periods. Unfortunately, we did not have deployment duration data that was specific to each capture and had to rely on fishery-wide averages or average durations provided by captains. Also, under these circumstances the natural dive duration before capture is unknown. Given these uncertainties, we were not able to confidently assess the effect of submergence duration on GE risk. However, the steeper slope of increasing occurrence of GE with depth for turtles caught in gillnets suggests that the longer average deployment of gillnets may cause greater risk of DCS for the same depth as compared with average trawling times. A direct relationship between risk of GE and both greater duration and depth of deployment would explain why we did not observe differences in overall incidence of GE between these two very dissimilar fisheries. As there are considerable differences in the submergence duration, ascent rate or depth of the gear within and between fisheries, future studies should assess how these factors may affect risk of GE and DCS outcome.

Another important consideration is our limited understanding of the physiological and behavioral mechanisms that prevent DCS and other pressure related illnesses in marine vertebrates. In sea turtles, it has been hypothesized that the muscular sphincters within the pulmonary arteries contract during diving, shunting blood away from the lungs^16, 20^. This response may prevent N_2_ uptake during the dive, thus minimizing the risk of DCS. However, sympathetic stimulation caused by a fight-or-flight response, as would happen upon capture, could alter shunting and increase N_2_ uptake. Consequently, the variation in the degree of excitation and exertion created during the capture event and individual responses could affect risk of DCS.

The only physiological variable that has been indisputably correlated with DCS risk in air breathing vertebrates is *M*_b_^1, 4, 5^. The theoretical basis for the correlation between organism mass and DCS risk lies in how inert gases are taken up and removed. Removal and uptake of metabolic gases are thought to be perfusion-limited^21^, and therefore variation in risk should correlate with changes in cardiac output. As cardiac output scales allometrically with *M*_b_, DCS risk also scales with *M*_b_^1^. As ectothermic animals, the metabolic rate and cardiac output of turtles depends on both animal size and ambient temperature^22^. We did not find evidence that either *M*_b_ or SST affected risk of GE, but acknowledge risk of type II error in our analyses. First, we used SST in lieu of unavailable subsurface temperatures, which are likely to decrease with depth and are more reflective of animal status upon capture. Nonetheless, we elected to include SST given the importance of water temperature reported in previous studies of post-interaction mortality^17–19^, and because turtles are exposed to SST after they are brought to surface and, if released, while they are developing GE. Another consideration is that larger turtles were caught at deeper depths, which may have confounded the results. These caveats highlight the challenges of working with opportunistic data without experimental control. The binary nature of DCS outcome (i.e., either yes or no) requires a large sample size to thoroughly investigate potential relationships, which is why experimental decompression studies closely control *M*_b_ and dive profile^1^. Despite our results, the influence of turtle size and water temperature on GE is still a worthwhile area for further study. In addition, the time course from surfacing until GE appears, and the time course of the disease will be an important area for future research as it may significantly alter outcome and long-term survival.

The correlation between GE and depth (Figs 2–4) of gear deployment is important because it further corroborates the occurrence of DCS in sea turtles subjected to underwater capture, and is a fundamental step towards improved understanding of this condition. There are additional potential variables that may also play a role in the occurrence of DCS that were not examinable based on the available data. In addition to those mentioned, rate of decompression (the rate at which nets are hauled out of the water) would be of particular interest given its importance in other taxa^1^, and the potential differences in this variable between fisheries. While our work suggests that depth alone significantly influences risk and severity of GE, efforts are needed to study DCS in other situations and obtain more detailed information on deployment parameters and environmental conditions associated with its occurrence. Identification of major risk factors may help develop fishing practices that minimize sea turtle mortality caused by decompression.

## Material and Methods

### Data acquisition and animal care permits

All data used in this study were provided by the Veterinary Department at Oceanogràfic, Valencia, Spain. All activities related to veterinary evaluation of bycaught turtles were conducted under an official signed agreement provided by the Government of the Valencia Region. The objective of these activities was to provide appropriate care and maximize survivorship. No procedures were conducted solely for research purposes. Depth of fishing gear deployment was obtained from information provided by fisherman to researchers at the time of turtle admission to the veterinary clinic. Sea surface temperature was estimated using the reported GPS position and data available on http://www.seatemperature.org/europe/spain/.

### Animals

Loggerhead turtles that were incidentally captured (bycaught) in trawls and gillnets along the coast of Eastern Spain were brought ashore for veterinary evaluation and treatment. All bycaught turtles from participating fishing boats were brought in, even if the animal did not show signs of disease or trauma. The duration from surfacing until the animal arrived at the veterinary clinic was not known. Examination included measurement of *M*_b_ (kg) and *CC*_L_ (cm, from mid-point of the nuchal scute to the posterior-most tip of the carapace^23^), physical visual examination, neurologic examination, routine hematology and blood chemistry analysis, ultrasonography of the coelomic cavity through standard acoustic windows (General Electric Logiq E Vet ultrasound machine, GE Medical Systems), and full-body radiographs in cranial-caudal, lateral-lateral (LL), and dorsal-ventral (DV) projections (Philips Practix 400 unit, Philips Medical Systems; and a Kodak Direct View Classic CR System, Carestream Health).

### Gas embolism (GE) score

The ultrasound and radiographs were used to assess presence (1) or absence (0) of GE, and to score the severity of the bubble formation on a scale of 1 to 5 using the criteria detailed in Fig. 1.

### Statistical analysis

Relationships between continuous variables were analyzed using ordinary least squares. Relationships between binary dependent variables (e.g., GE) and other variables were analyzed using logistic regression with the logit link. Ordinal regression with the logit link was used to separate the risk of DCS based on GE scores. All models were assessed for goodness of fit using both graphical and quantitative means and were found to be adequate.

95% confidence intervals for the probability of gas emboli were plotted using a non-linear parameter estimation routine written for the Matlab language^24^ and using propagation of error formulas^25^.

In this study P-values ≤ 0.05 were considered as significant and P ≤ 0.1 were considered a trend. Data are presented as the mean ± standard deviation (s.d.), unless otherwise stated.

All statistical calculations were performed using the statistical language R^26^, including the ordinal package^27^.

### Data availability

The R-code and data sets used in this study are freely available at the following link osf.io/5t9qg.
